# 5-Methyl-12-phenyl­sulfonyl-12*H*-naphtho­[1,2-*b*]carbazole

**DOI:** 10.1107/S160053681104949X

**Published:** 2011-11-30

**Authors:** S. Vasudhevan, R. Joel Karunakaran

**Affiliations:** a Department of Chemistry, Madras Christian College (Autonomous), Chennai 600 059, Tamil Nadu, India

## Abstract

In the title compound, C_27_H_19_NO_2_S, the naphtho­carbazole unit is approximately planar (r.m.s. deviation = 0.002 Å) except for the N atom, which is displaced by 0.122 (1) Å out of the mean plane. The dihedral angle between the naphtho­carbazole mean plane and the phenyl ring of the phenyl­sulfonyl substituent is 83.16 (3)°. An inter­molecular C—H⋯π inter­action involving the phenyl group and the pyrrole ring is observed in the crystal structure.

## Related literature

For the biological activity of indole and carbazole derivatives see: Chai *et al.* (2006 ▶); Rani *et al.* (2004 ▶); Panwar *et al.* (2006 ▶); Abele *et al.* (2003 ▶). For related structures see: Chakkaravarthi *et al.* (2007 ▶); Liu *et al.* (2007 ▶).
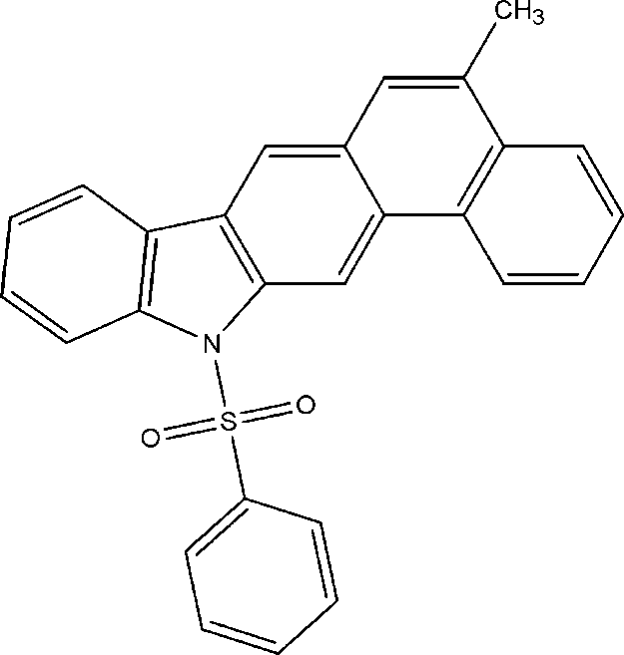


## Experimental

### 

#### Crystal data

C_27_H_19_NO_2_S
*M* *_r_* = 421.49
Triclinic, 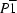

*a* = 9.4527 (3) Å
*b* = 10.7457 (3) Å
*c* = 11.5791 (3) Å
α = 115.592 (1)°
β = 93.324 (2)°
γ = 105.206 (2)°
*V* = 1003.61 (5) Å^3^
*Z* = 2
Mo *K*α radiation
μ = 0.19 mm^−1^
*T* = 295 K
0.30 × 0.25 × 0.20 mm

#### Data collection

Bruker Kappa APEXII CCD diffractometer
Absorption correction: multi-scan (*SADABS*; Sheldrick, 1996 ▶) *T*_min_ = 0.946, *T*_max_ = 0.964
23737 measured reflections
5360 independent reflections
4477 reflections with *I* > 2σ(*I*)
*R*_int_ = 0.027

#### Refinement

*R*[*F* ^2^ > 2σ(*F* ^2^)] = 0.039
*wR*(*F* ^2^) = 0.120
*S* = 1.05
5360 reflections
281 parameters
H-atom parameters constrained
Δρ_max_ = 0.38 e Å^−3^
Δρ_min_ = −0.29 e Å^−3^

### 

Data collection: *APEX2* (Bruker, 2004 ▶); cell refinement: *SAINT* (Bruker, 2004 ▶); data reduction: *SAINT*; program(s) used to solve structure: *SHELXS97* (Sheldrick, 2008 ▶); program(s) used to refine structure: *SHELXL97* (Sheldrick, 2008 ▶); molecular graphics: *PLATON* (Spek, 2009 ▶); software used to prepare material for publication: *SHELXL97*.

## Supplementary Material

Crystal structure: contains datablock(s) I, global. DOI: 10.1107/S160053681104949X/bh2396sup1.cif

Structure factors: contains datablock(s) I. DOI: 10.1107/S160053681104949X/bh2396Isup2.hkl

Supplementary material file. DOI: 10.1107/S160053681104949X/bh2396Isup3.cml

Additional supplementary materials:  crystallographic information; 3D view; checkCIF report

## Figures and Tables

**Table 1:** Hydrogen-bond geometry (Å, °) *Cg*1 is the centroid of the pyrrole ring.

*D*—H⋯*A*	*D*—H	H⋯*A*	*D*⋯*A*	*D*—H⋯*A*
C25—H25⋯*Cg*1^i^	0.93	2.61	3.4770 (2)	156

Symmetry code: (i) 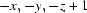.

**Fig. 1.**
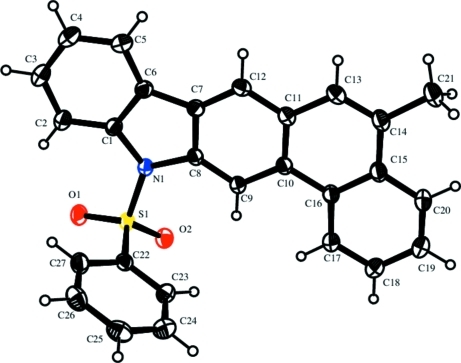
The molecular structure of the title compound, with 30% probability displacement ellipsoids for non-H atoms.

## supplementary crystallographic information

### Comment

Heterocyclic compounds containing pyrrole ring with benzene ring fused to *α*,*β*-position with π electrons and lone pair from the nitrogen atom are biologically accepted pharmacophore in medicinal compounds and possesses a wide spectrum of biological activities (Chai *et al.*, 2006). Biological activity includes anticancer, anti-inflammatory (Rani *et al.*, 2004), analgesic, antimicrobial (Panwar *et al.*, 2006) and antifungal (Abele *et al.*, 2003) properties.

The geometric parameters of the title molecule (Fig. 1) agree well with reported similar structures (Chakkaravarthi *et al.*, 2007; Liu *et al.*, 2007). The naphtho-carbazole moiety is planar except that the nitrogen atom N1 is 0.122 Å out of the mean plane. Dihedral angle between the mean plane of the naphtho-carbazole moiety and the phenyl group is 83.16 (3)°. The geometry of bonding of sulfur atom is tetrahedral except that angle O1—S1—O2 is 120.40 (6)°. There is a C—H···π interaction between the five membered ring (C1, C6, C7, C8 and N1) and H25^i^ (symmetry code: i = -*x*, -*y*, 1 - *z*) of the phenyl ring. The separation between the H atom and the centroid of the five membered ring is 2.61 Å.

### Experimental

To a solution of diethyl-2-((bromomethyl-1-(phenylsulfonyl)-1*H*- indole-3-yl)methylene)malonate (0.2 g, 0.38 mmol) in dry 1,2-DCE (10 ml), ZnBr_2_ (0.17 g, 0.75 mmol) and 1-methylnaphthalene (0.06 ml, 0.42 mmol) were added. The reaction mixture was then refluxed for 1 h under nitrogen atmosphere. It was then poured over ice-water (50 ml) containing 2 ml of conc. HCl, extracted with chloroform (3 × 10 ml) and dried over Na_2_SO_4_. The removal of solvent followed by flash column chromatographic purification (silica gel, 230–420 mesh, *n*-hexane/ethyl acetate 96:4) afforded the title carbazole as a colourless solid. Recrystallization was done using CDCl_3_ as solvent.

### Refinement

H atoms were positioned geometrically and refined using the riding model with C—H = 0.93 Å and *U*_iso_(H) = 1.2*U*_eq_(C) for aromatic CH, and C—H = 0.96 Å and *U*_iso_(H) = 1.5*U*_eq_(C) for the methyl group.

### Crystal data

**Table:**

C_27_H_19_NO_2_S	*Z* = 2
*M**_r_* = 421.49	*F*(000) = 440
Triclinic, *P*1	*D*_x_ = 1.395 Mg m^−^^3^
Hall symbol: -P 1	Mo *K*α radiation, λ = 0.71073 Å
*a* = 9.4527 (3) Å	Cell parameters from 6046 reflections
*b* = 10.7457 (3) Å	θ = 2.2–29.0°
*c* = 11.5791 (3) Å	µ = 0.19 mm^−^^1^
α = 115.592 (1)°	*T* = 295 K
β = 93.324 (2)°	Prism, colourless
γ = 105.206 (2)°	0.30 × 0.25 × 0.20 mm
*V* = 1003.61 (5) Å^3^	

### Data collection

**Table:**

Bruker Kappa APEXII CCD diffractometer	5360 independent reflections
Radiation source: fine-focus sealed tube	4477 reflections with *I* > 2σ(*I*)
graphite	*R*_int_ = 0.027
Detector resolution: 0 pixels mm^-1^	θ_max_ = 29.1°, θ_min_ = 2.2°
ω and φ scans	*h* = −12→12
Absorption correction: multi-scan (*SADABS*; Sheldrick, 1996)	*k* = −14→14
*T*_min_ = 0.946, *T*_max_ = 0.964	*l* = −15→15
23737 measured reflections	

### Refinement

**Table:**

Refinement on *F*^2^	Primary atom site location: structure-invariant direct methods
Least-squares matrix: full	Secondary atom site location: difference Fourier map
*R*[*F*^2^ > 2σ(*F*^2^)] = 0.039	Hydrogen site location: inferred from neighbouring sites
*wR*(*F*^2^) = 0.120	H-atom parameters constrained
*S* = 1.05	*w* = 1/[σ^2^(*F*_o_^2^) + (0.0679*P*)^2^ + 0.1767*P*] where *P* = (*F*_o_^2^ + 2*F*_c_^2^)/3
5360 reflections	(Δ/σ)_max_ < 0.001
281 parameters	Δρ_max_ = 0.38 e Å^−^^3^
0 restraints	Δρ_min_ = −0.29 e Å^−^^3^

### Fractional atomic coordinates and isotropic or equivalent isotropic displacement parameters (Å^2^)

**Table:**

	*x*	*y*	*z*	*U*_iso_*/*U*_eq_	
C1	0.18888 (14)	0.24807 (14)	0.29115 (12)	0.0353 (3)	
C2	0.09995 (18)	0.17091 (17)	0.16743 (14)	0.0485 (3)	
H2	0.1056	0.0812	0.1079	0.058*	
C3	0.0023 (2)	0.2323 (2)	0.13579 (16)	0.0581 (4)	
H3	−0.0599	0.1819	0.0538	0.070*	
C4	−0.0052 (2)	0.3660 (2)	0.22221 (17)	0.0591 (4)	
H4	−0.0720	0.4043	0.1979	0.071*	
C5	0.08503 (18)	0.44334 (17)	0.34419 (16)	0.0502 (4)	
H5	0.0808	0.5344	0.4020	0.060*	
C6	0.18221 (15)	0.38411 (14)	0.37986 (13)	0.0362 (3)	
C7	0.28394 (14)	0.43405 (13)	0.50043 (12)	0.0338 (3)	
C8	0.35218 (13)	0.32723 (12)	0.48302 (11)	0.0316 (2)	
C9	0.45294 (14)	0.34082 (13)	0.58145 (12)	0.0346 (3)	
H9	0.4977	0.2694	0.5675	0.042*	
C10	0.48713 (14)	0.46487 (13)	0.70383 (12)	0.0331 (3)	
C11	0.42165 (15)	0.57442 (13)	0.72159 (12)	0.0363 (3)	
C12	0.32029 (15)	0.55734 (14)	0.61848 (13)	0.0389 (3)	
H12	0.2776	0.6296	0.6300	0.047*	
C13	0.46254 (17)	0.70407 (14)	0.84397 (14)	0.0444 (3)	
H13	0.4194	0.7756	0.8538	0.053*	
C14	0.56095 (17)	0.72673 (15)	0.94549 (13)	0.0447 (3)	
C15	0.62605 (16)	0.61556 (14)	0.93291 (12)	0.0404 (3)	
C16	0.59082 (15)	0.48544 (13)	0.81357 (12)	0.0367 (3)	
C17	0.65618 (19)	0.38032 (16)	0.80598 (14)	0.0493 (4)	
H17	0.6333	0.2943	0.7283	0.059*	
C18	0.7536 (2)	0.40075 (19)	0.91058 (16)	0.0605 (4)	
H18	0.7960	0.3294	0.9032	0.073*	
C19	0.7880 (2)	0.52832 (19)	1.02674 (16)	0.0625 (5)	
H19	0.8538	0.5427	1.0976	0.075*	
C20	0.7258 (2)	0.63266 (17)	1.03758 (14)	0.0535 (4)	
H20	0.7499	0.7176	1.1164	0.064*	
C21	0.6035 (2)	0.86763 (18)	1.07059 (16)	0.0640 (5)	
H21A	0.5506	0.9292	1.0621	0.096*	
H21B	0.7091	0.9158	1.0881	0.096*	
H21C	0.5777	0.8477	1.1412	0.096*	
C22	0.21436 (14)	−0.03734 (13)	0.37019 (12)	0.0345 (3)	
C23	0.27678 (16)	−0.03293 (15)	0.48342 (14)	0.0416 (3)	
H23	0.3771	0.0189	0.5212	0.050*	
C24	0.18862 (19)	−0.10623 (18)	0.53972 (16)	0.0515 (4)	
H24	0.2293	−0.1045	0.6157	0.062*	
C25	0.04021 (19)	−0.18190 (17)	0.48296 (17)	0.0540 (4)	
H25	−0.0191	−0.2308	0.5214	0.065*	
C26	−0.02169 (18)	−0.18632 (17)	0.37012 (17)	0.0535 (4)	
H26	−0.1222	−0.2380	0.3329	0.064*	
C27	0.06510 (16)	−0.11417 (15)	0.31198 (14)	0.0449 (3)	
H27	0.0243	−0.1170	0.2355	0.054*	
N1	0.29778 (12)	0.21380 (11)	0.35213 (10)	0.0351 (2)	
O1	0.26995 (12)	−0.02036 (11)	0.16013 (9)	0.0458 (2)	
O2	0.47830 (10)	0.08335 (10)	0.34758 (9)	0.0414 (2)	
S1	0.32587 (3)	0.05452 (3)	0.29722 (3)	0.03372 (10)	

### Atomic displacement parameters (Å^2^)

**Table:**

	*U*^11^	*U*^22^	*U*^33^	*U*^12^	*U*^13^	*U*^23^
C1	0.0346 (6)	0.0376 (6)	0.0340 (6)	0.0090 (5)	0.0029 (5)	0.0189 (5)
C2	0.0535 (9)	0.0477 (8)	0.0375 (7)	0.0172 (7)	−0.0048 (6)	0.0147 (6)
C3	0.0605 (10)	0.0640 (10)	0.0474 (8)	0.0204 (8)	−0.0091 (7)	0.0258 (8)
C4	0.0613 (10)	0.0668 (10)	0.0589 (10)	0.0291 (8)	−0.0019 (8)	0.0345 (8)
C5	0.0558 (9)	0.0471 (8)	0.0532 (9)	0.0225 (7)	0.0039 (7)	0.0254 (7)
C6	0.0364 (6)	0.0360 (6)	0.0380 (6)	0.0095 (5)	0.0049 (5)	0.0201 (5)
C7	0.0328 (6)	0.0319 (6)	0.0362 (6)	0.0075 (5)	0.0052 (5)	0.0173 (5)
C8	0.0317 (6)	0.0284 (5)	0.0292 (5)	0.0056 (4)	0.0040 (4)	0.0111 (4)
C9	0.0365 (6)	0.0309 (6)	0.0312 (6)	0.0100 (5)	0.0020 (5)	0.0107 (5)
C10	0.0344 (6)	0.0300 (5)	0.0302 (6)	0.0063 (5)	0.0048 (4)	0.0121 (5)
C11	0.0382 (7)	0.0309 (6)	0.0338 (6)	0.0081 (5)	0.0064 (5)	0.0116 (5)
C12	0.0419 (7)	0.0319 (6)	0.0419 (7)	0.0142 (5)	0.0064 (5)	0.0151 (5)
C13	0.0519 (8)	0.0327 (6)	0.0407 (7)	0.0150 (6)	0.0076 (6)	0.0096 (5)
C14	0.0512 (8)	0.0364 (6)	0.0337 (6)	0.0094 (6)	0.0062 (6)	0.0079 (5)
C15	0.0456 (7)	0.0355 (6)	0.0307 (6)	0.0058 (5)	0.0041 (5)	0.0116 (5)
C16	0.0397 (7)	0.0335 (6)	0.0306 (6)	0.0067 (5)	0.0031 (5)	0.0125 (5)
C17	0.0613 (9)	0.0408 (7)	0.0372 (7)	0.0178 (7)	−0.0031 (6)	0.0113 (6)
C18	0.0766 (12)	0.0516 (9)	0.0485 (9)	0.0251 (8)	−0.0101 (8)	0.0191 (7)
C19	0.0790 (12)	0.0561 (9)	0.0422 (8)	0.0184 (8)	−0.0139 (8)	0.0187 (7)
C20	0.0673 (10)	0.0451 (8)	0.0328 (7)	0.0099 (7)	−0.0054 (6)	0.0111 (6)
C21	0.0805 (12)	0.0458 (8)	0.0424 (8)	0.0222 (8)	−0.0008 (8)	0.0005 (7)
C22	0.0339 (6)	0.0299 (5)	0.0355 (6)	0.0112 (5)	0.0042 (5)	0.0114 (5)
C23	0.0374 (7)	0.0442 (7)	0.0436 (7)	0.0138 (6)	0.0035 (5)	0.0208 (6)
C24	0.0567 (9)	0.0550 (8)	0.0525 (9)	0.0209 (7)	0.0130 (7)	0.0315 (7)
C25	0.0565 (9)	0.0440 (8)	0.0647 (10)	0.0143 (7)	0.0239 (8)	0.0276 (7)
C26	0.0391 (8)	0.0444 (8)	0.0594 (9)	0.0033 (6)	0.0065 (7)	0.0150 (7)
C27	0.0390 (7)	0.0409 (7)	0.0434 (7)	0.0080 (6)	−0.0013 (6)	0.0134 (6)
N1	0.0378 (6)	0.0323 (5)	0.0290 (5)	0.0108 (4)	−0.0012 (4)	0.0101 (4)
O1	0.0552 (6)	0.0442 (5)	0.0280 (5)	0.0177 (4)	0.0019 (4)	0.0078 (4)
O2	0.0335 (5)	0.0459 (5)	0.0406 (5)	0.0156 (4)	0.0065 (4)	0.0150 (4)
S1	0.03463 (17)	0.03321 (16)	0.02764 (16)	0.01181 (12)	0.00277 (11)	0.00900 (12)

### Geometric parameters (Å, °)

**Table:**

C1—C2	1.3838 (18)	C15—C16	1.4172 (17)
C1—C6	1.3981 (18)	C16—C17	1.397 (2)
C1—N1	1.4258 (16)	C17—C18	1.378 (2)
C2—C3	1.382 (2)	C17—H17	0.9300
C2—H2	0.9300	C18—C19	1.384 (2)
C3—C4	1.374 (3)	C18—H18	0.9300
C3—H3	0.9300	C19—C20	1.361 (2)
C4—C5	1.374 (2)	C19—H19	0.9300
C4—H4	0.9300	C20—H20	0.9300
C5—C6	1.3818 (19)	C21—H21A	0.9600
C5—H5	0.9300	C21—H21B	0.9600
C6—C7	1.4451 (17)	C21—H21C	0.9600
C7—C12	1.3725 (17)	C22—C23	1.3825 (18)
C7—C8	1.4063 (17)	C22—C27	1.3872 (18)
C8—C9	1.3729 (16)	C22—S1	1.7542 (13)
C8—N1	1.4255 (14)	C23—C24	1.379 (2)
C9—C10	1.4065 (16)	C23—H23	0.9300
C9—H9	0.9300	C24—C25	1.376 (2)
C10—C11	1.4139 (18)	C24—H24	0.9300
C10—C16	1.4572 (17)	C25—C26	1.377 (2)
C11—C12	1.4010 (18)	C25—H25	0.9300
C11—C13	1.4307 (18)	C26—C27	1.382 (2)
C12—H12	0.9300	C26—H26	0.9300
C13—C14	1.347 (2)	C27—H27	0.9300
C13—H13	0.9300	N1—S1	1.6499 (11)
C14—C15	1.440 (2)	O1—S1	1.4223 (9)
C14—C21	1.5069 (19)	O2—S1	1.4228 (10)
C15—C20	1.406 (2)		
			
C2—C1—C6	121.28 (12)	C15—C16—C10	119.28 (12)
C2—C1—N1	130.60 (12)	C18—C17—C16	121.74 (14)
C6—C1—N1	108.12 (11)	C18—C17—H17	119.1
C3—C2—C1	117.42 (14)	C16—C17—H17	119.1
C3—C2—H2	121.3	C17—C18—C19	119.51 (16)
C1—C2—H2	121.3	C17—C18—H18	120.2
C4—C3—C2	121.79 (15)	C19—C18—H18	120.2
C4—C3—H3	119.1	C20—C19—C18	120.24 (15)
C2—C3—H3	119.1	C20—C19—H19	119.9
C5—C4—C3	120.61 (15)	C18—C19—H19	119.9
C5—C4—H4	119.7	C19—C20—C15	121.79 (14)
C3—C4—H4	119.7	C19—C20—H20	119.1
C4—C5—C6	119.13 (15)	C15—C20—H20	119.1
C4—C5—H5	120.4	C14—C21—H21A	109.5
C6—C5—H5	120.4	C14—C21—H21B	109.5
C5—C6—C1	119.74 (13)	H21A—C21—H21B	109.5
C5—C6—C7	132.16 (13)	C14—C21—H21C	109.5
C1—C6—C7	108.06 (11)	H21A—C21—H21C	109.5
C12—C7—C8	119.46 (11)	H21B—C21—H21C	109.5
C12—C7—C6	132.79 (12)	C23—C22—C27	121.37 (13)
C8—C7—C6	107.75 (11)	C23—C22—S1	119.60 (10)
C9—C8—C7	122.08 (11)	C27—C22—S1	119.03 (10)
C9—C8—N1	129.99 (11)	C24—C23—C22	119.25 (13)
C7—C8—N1	107.94 (10)	C24—C23—H23	120.4
C8—C9—C10	118.68 (12)	C22—C23—H23	120.4
C8—C9—H9	120.7	C25—C24—C23	119.69 (15)
C10—C9—H9	120.7	C25—C24—H24	120.2
C9—C10—C11	119.69 (11)	C23—C24—H24	120.2
C9—C10—C16	121.78 (12)	C24—C25—C26	120.97 (15)
C11—C10—C16	118.52 (11)	C24—C25—H25	119.5
C12—C11—C10	119.97 (12)	C26—C25—H25	119.5
C12—C11—C13	120.35 (12)	C25—C26—C27	120.14 (14)
C10—C11—C13	119.66 (12)	C25—C26—H26	119.9
C7—C12—C11	120.07 (12)	C27—C26—H26	119.9
C7—C12—H12	120.0	C26—C27—C22	118.58 (14)
C11—C12—H12	120.0	C26—C27—H27	120.7
C14—C13—C11	122.69 (14)	C22—C27—H27	120.7
C14—C13—H13	118.7	C8—N1—C1	108.03 (10)
C11—C13—H13	118.7	C8—N1—S1	124.03 (8)
C13—C14—C15	119.35 (12)	C1—N1—S1	126.23 (8)
C13—C14—C21	120.28 (14)	O1—S1—O2	120.40 (6)
C15—C14—C21	120.37 (14)	O1—S1—N1	106.72 (6)
C20—C15—C16	118.27 (14)	O2—S1—N1	106.51 (6)
C20—C15—C14	121.30 (13)	O1—S1—C22	108.78 (6)
C16—C15—C14	120.43 (12)	O2—S1—C22	108.04 (6)
C17—C16—C15	118.46 (12)	N1—S1—C22	105.42 (6)
C17—C16—C10	122.26 (12)		
			
C6—C1—C2—C3	−1.1 (2)	C20—C15—C16—C10	179.69 (13)
N1—C1—C2—C3	179.49 (15)	C14—C15—C16—C10	−0.39 (19)
C1—C2—C3—C4	1.1 (3)	C9—C10—C16—C17	−3.4 (2)
C2—C3—C4—C5	−0.1 (3)	C11—C10—C16—C17	177.57 (13)
C3—C4—C5—C6	−1.0 (3)	C9—C10—C16—C15	177.25 (12)
C4—C5—C6—C1	0.9 (2)	C11—C10—C16—C15	−1.77 (18)
C4—C5—C6—C7	−176.62 (15)	C15—C16—C17—C18	−0.4 (2)
C2—C1—C6—C5	0.1 (2)	C10—C16—C17—C18	−179.74 (15)
N1—C1—C6—C5	179.63 (13)	C16—C17—C18—C19	0.2 (3)
C2—C1—C6—C7	178.23 (13)	C17—C18—C19—C20	0.1 (3)
N1—C1—C6—C7	−2.27 (14)	C18—C19—C20—C15	−0.2 (3)
C5—C6—C7—C12	−1.4 (3)	C16—C15—C20—C19	−0.1 (2)
C1—C6—C7—C12	−179.23 (14)	C14—C15—C20—C19	−179.97 (16)
C5—C6—C7—C8	178.09 (15)	C27—C22—C23—C24	−0.1 (2)
C1—C6—C7—C8	0.31 (14)	S1—C22—C23—C24	−179.57 (11)
C12—C7—C8—C9	1.12 (19)	C22—C23—C24—C25	−0.3 (2)
C6—C7—C8—C9	−178.49 (11)	C23—C24—C25—C26	0.4 (2)
C12—C7—C8—N1	−178.62 (11)	C24—C25—C26—C27	0.0 (3)
C6—C7—C8—N1	1.77 (13)	C25—C26—C27—C22	−0.4 (2)
C7—C8—C9—C10	0.81 (19)	C23—C22—C27—C26	0.5 (2)
N1—C8—C9—C10	−179.52 (12)	S1—C22—C27—C26	179.90 (11)
C8—C9—C10—C11	−2.16 (18)	C9—C8—N1—C1	177.12 (12)
C8—C9—C10—C16	178.83 (11)	C7—C8—N1—C1	−3.17 (13)
C9—C10—C11—C12	1.62 (19)	C9—C8—N1—S1	11.21 (19)
C16—C10—C11—C12	−179.33 (12)	C7—C8—N1—S1	−169.08 (9)
C9—C10—C11—C13	−176.69 (12)	C2—C1—N1—C8	−177.19 (14)
C16—C10—C11—C13	2.35 (19)	C6—C1—N1—C8	3.37 (14)
C8—C7—C12—C11	−1.66 (19)	C2—C1—N1—S1	−11.7 (2)
C6—C7—C12—C11	177.83 (13)	C6—C1—N1—S1	168.89 (9)
C10—C11—C12—C7	0.3 (2)	C8—N1—S1—O1	−173.31 (10)
C13—C11—C12—C7	178.62 (12)	C1—N1—S1—O1	23.37 (13)
C12—C11—C13—C14	−179.06 (14)	C8—N1—S1—O2	−43.51 (11)
C10—C11—C13—C14	−0.7 (2)	C1—N1—S1—O2	153.16 (11)
C11—C13—C14—C15	−1.5 (2)	C8—N1—S1—C22	71.12 (11)
C11—C13—C14—C21	178.00 (14)	C1—N1—S1—C22	−92.20 (11)
C13—C14—C15—C20	−178.06 (15)	C23—C22—S1—O1	150.21 (11)
C21—C14—C15—C20	2.5 (2)	C27—C22—S1—O1	−29.23 (13)
C13—C14—C15—C16	2.0 (2)	C23—C22—S1—O2	17.93 (13)
C21—C14—C15—C16	−177.44 (14)	C27—C22—S1—O2	−161.51 (11)
C20—C15—C16—C17	0.3 (2)	C23—C22—S1—N1	−95.64 (11)
C14—C15—C16—C17	−179.76 (14)	C27—C22—S1—N1	84.91 (11)

### Hydrogen-bond geometry (Å, °)

**Table:**

Cg1 is the centroid of the pyrrole ring.

**Table:**

*D*—H···*A*	*D*—H	H···*A*	*D*···*A*	*D*—H···*A*
C2—H2···O1	0.93	2.31	2.9003 (18)	121.
C9—H9···O2	0.93	2.42	2.9994 (15)	120.
C23—H23···O2	0.93	2.55	2.9088 (18)	103.
C25—H25···Cg1^i^	0.93	2.61	3.4770 (2)	156.

Symmetry codes: (i) −*x*, −*y*, −*z*+1.
